# Supplementary material for: Phenotypes of painful TMD in discordant monozygotic twins according to a cognitive-behavioral-emotional model: a case-control study
Source: PLoS One. 2025 Apr 17;20(4):e0320515. doi: 10.1371/journal.pone.0320515 (PMC12005551; doi:10.1371/journal.pone.0320515)
Supplement: Attachment — Submitted filename: Response to Reviewers.docx [file pone.0320515.s002.docx]

**Reviewer #1	Manuscript PONE-D-24-27113**

**General comments**

*The topic of the study is both interesting and well-defined. However, the discussion section could benefit from further refinement.*

**Response:** We would like to thank you for your detailed analysis of our manuscript. Below, we present clarifications of the points raised, and the manuscript has been reformulated based on the suggestions made. We believe the revised version has been improved, and we would like a new manuscript analysis.

**Specific comments**

1. *In the first paragraph of the introduction, it would be beneficial to better describe the specificity of the TMD diagnoses. For instance, the study focuses specifically on painful TMD, a point that could be highlighted earlier in the introduction.*

**Response:** We appreciate the reviewer’s insightful suggestion. To address this, we have revised the first paragraph of the introduction to emphasize the specific focus on painful temporomandibular disorders (TMD) earlier in the text and added a second sentence about TMD diagnosis. We have clarified that the study specifically targets painful TMD, which allows for a more precise understanding of the population under investigation. This revision clarifies the study’s objectives and scope from the outset. The revised sentences are described below.

Page 3, lines 2 to 19 revised version:

“Temporomandibular disorders (TMD), specifically those associated with pain, are currently understood as a set of signs and symptoms that designate a painful musculoskeletal syndrome, often linked to multisystemic changes, including alterations in behavior, emotional status, and social interactions. These manifestations are recognized as expressions of central nervous system dysregulation. Among the main predictors for the development of painful TMD are the presence of comorbidities, non-painful orofacial symptoms (such as self-reported parafunctions), the frequency of somatic symptoms, poor sleep quality, and genetic and epigenetic factors. This study focuses on the painful subtype of TMD, which is of particular clinical relevance due to its impact on quality of life and its association with chronic pain mechanisms [1, 2].

Painful TMD includes diagnoses such as myofascial pain, arthralgia, and headaches attributed to TMD, all of which are characterized by chronic orofacial pain and functional limitations. These painful subtypes differ from intra-articular TMD, which may present with joint noises or movement restrictions without significant painful discomfort. The specificity of diagnosing painful TMD is guided by the Diagnostic Criteria for Temporomandibular Disorders (DC/TMD), which allows for the accurate identification of patients experiencing TMD for the last month. By focusing on painful TMD, this study aims to explore the cognitive-behavioral-emotional factors contributing to the onset and maintenance of pain in this specific population, distinguishing it from the broader spectrum of TMD presentations [3].”

2. *The methodology is robust and well-detailed. The statistical analyses and results are presented clearly and concisely. It would be helpful to explicitly mention which variables were excluded due to collinearity during the regression model formation. While this is somewhat implied, providing this information would aid in the reader’s interpretation.*

**Response:** We appreciate the reviewer’s positive feedback on the methodology and the suggestion to improve the clarity of the statistical analyses. In response, we have revised the manuscript to explicitly mention which variables were excluded due to collinearity during the formation of the regression model. Specifically, the final model did not include variables that showed significant collinearity, as indicated by Spearman's correlation coefficient (p < 0.05). This information has been added to the "Statistical Analyses" section to provide readers with a clearer understanding of the model selection process. The added sentences are described below.

Page 8, lines 20 to 31, and page 9, lines 1 to 12

“During the development of the multiple linear regression model, certain variables were excluded due to significant collinearity to maintain the model’s robustness and ensure accurate and reliable estimates. Collinearity was identified using Spearman's correlation coefficient, with variables showing a p-value < 0.05 being excluded from the final model to avoid multicollinearity, which could distort the relationships between the predictors and the outcome. The excluded variables include right upper trapezius pain threshold (OR=0.29, 95%CI=0.07–1.20, p=0.088), right temporomandibular joint (TMJ) pain sensitivity (OR=1.15, 95%CI=0.86–1.54, p=0.346), and left masseter pain threshold (OR=2.28, 95%CI=0.82–6.31, p=0.113). Although these variables showed associations with TMD in the bivariate analysis, their high correlation with other variables in the model necessitated their exclusion. Similarly, sleep quality total score (OR=1.60, 95%CI=0.88–2.93, p=0.124), pain awareness total score (OR=1.07, 95%CI=0.98–1.16, p=0.158), and daytime oral behavior score (OR=1.33, 95%CI=0.88–2.02, p=0.180) were also excluded due to collinearity. Pain vigilance score (OR=1.14, 95%CI=0.95–1.37, p=0.154) significantly correlated with other variables and was similarly removed from the final model.

By excluding these collinear variables, we retained only those predictors that demonstrated no significant collinearity, thus ensuring the independence of the variables in the regression model. The retained variables, including left masseter pain sensitivity, total pain catastrophizing score, difficulty in externalizing feelings, and the distraction facet of the mindfulness scale, provided a more precise and accurate representation of the factors associated with the development of painful TMD. This step was essential for enhancing the model's validity and ensuring that the conclusions drawn are robust and meaningful.”

3. *The discussion section is somewhat lengthy and does not thoroughly explore the study’s findings. Previous studies could be summarized more concisely and directly linked to your results.*

**Response:** We appreciate the reviewer’s valuable feedback. In response to this comment, we have revised the discussion section to be more concise and to provide a more explicit focus on the study's findings. We have summarized previous studies more succinctly and emphasized direct connections between our results and the existing literature. Specifically, we have reduced the length of background information and ensured that comparisons with previous research are more clearly tied to the cognitive-behavioral-emotional model explored in our study. Furthermore, we have restructured the discussion to highlight our key findings regarding the role of pain sensitivity, catastrophizing, difficulty in externalizing feelings, and mindfulness distraction in predicting painful TMD. This revision enhances the clarity and relevance of the discussion, ensuring that the findings are thoroughly explored and linked to the broader context of temporomandibular disorder research. We believe these changes address the reviewer’s concern and improve the overall quality of the discussion.

4. *I suggest placing greater emphasis on the aspects related to epigenetics, the specificity of your sample, and the extensive number of variables investigated to construct the model—these are significant strengths of your study.*

**Response:** We appreciate the reviewer’s valuable suggestion to highlight the strengths of our study related to epigenetics, sample specificity, and the breadth of variables investigated. In response, we have revised the discussion to emphasize these aspects more. Specifically, we expanded the discussion on the role of epigenetic mechanisms in chronic pain and TMD, underscoring how the dynamic interaction between genetic predisposition and environmental factors may influence pain perception and susceptibility. The use of a monozygotic twin sample is a crucial strength of this study, as it allows us to minimize genetic variability, thus providing a unique opportunity to investigate the influence of non-genetic factors, including cognitive-behavioral-emotional aspects and epigenetic modifications, in the development of painful TMD.

Additionally, we have emphasized the extensive range of variables included in our analysis, which contributes to a more comprehensive understanding of the multifactorial nature of TMD. The breadth of variables investigated in this study, encompassing pain sensitivity, emotional regulation, mindfulness, and catastrophizing, allowed us to develop a robust model that reflects the complexity of the condition. These additions to the discussion underscore the strengths of our methodology and further highlight the significance of our findings.

5. *Another aspect that could be more thoroughly discussed is the idea that in genetically identical individuals, other factors may play a more significant role than those typically identified in genetically diverse populations.*

**Response:** We appreciate the reviewer’s thoughtful suggestion and have expanded the discussion to explore how, in genetically identical individuals, non-genetic factors such as environmental, psychological, and epigenetic influences may have a more significant impact than populations with greater genetic diversity. The use of monozygotic twins in our study allows for isolating these non-genetic influences, which provides a unique opportunity to investigate how cognitive-behavioral-emotional factors can override or modulate genetic predispositions. We have added a more detailed discussion on this point, emphasizing the growing evidence that even without genetic variability, psychological factors such as pain catastrophizing, emotional regulation, and mindfulness can play a critical role in pain perception and chronicity. This underscores the importance of focusing on these modifiable factors, which can be targeted in personalized interventions. The sentence below was added to the Discussion Section.

Page 17, lines 15 to 27

“In genetically identical individuals, such as the monozygotic twins in our study, non-genetic factors- environmental, psychological, and epigenetic- emerge as particularly influential in the development of chronic pain conditions like TMD. Our findings reinforce that, even without genetic diversity, cognitive-behavioral-emotional factors such as pain catastrophizing, difficulty in emotional expression, and mindfulness play a pivotal role in pain perception and modulation. Studies have shown that environmental stressors and psychological variables can induce epigenetic changes, influencing gene expression and central sensitization mechanisms [23,24]. This highlights the fact that in genetically identical populations, modifiable factors like emotional regulation and cognitive patterns may have a stronger impact on pain outcomes than genetic predispositions alone. The ability to isolate these influences in monozygotic twins underscores the importance of focusing on psychological and behavioral interventions, which may offer more targeted and effective treatments for chronic pain in individuals with similar genetic backgrounds.”

6. *The conclusion could be more concise and more precise.*

**Response:** Thank you for your suggestion. We have revised the conclusion to ensure it is more concise and focused. The revised version now directly summarizes the study's key findings, emphasizing the significant role of cognitive-behavioral-emotional factors in developing painful TMD while highlighting the potential for targeted interventions. We believe this revised conclusion is more straightforward and more aligned with the overall goals of the manuscript.

**Minor points**

1. **Note: * On page 12, in Table 3, the label for G7 is missing a "T."*

**Response:** Thank you for pointing out this error. We have corrected the label for G7 in Table 3 by adding the missing "T." The updated table now accurately reflects the correct labeling.

**Reviewer #2	Manuscript PONE-D-24-27113**

**General comments**

*Phenotypes of painful TMD in discordant monozygotic twins according to a cognitive behavioral-emotional model: a case-control study. It was described that the objective of this study “was to investigate variables based on a cognitive-behavioral-emotional model related to the development of painful temporomandibular disorders (TMD) in a sample of monozygotic twins discordant for the condition.”*

**Response:** We would like to thank you for your detailed analysis of our manuscript. Below, we present clarifications of the points raised, and the manuscript has been reformulated based on the suggestions made. We believe the revised version has been improved, and we would like a new manuscript analysis.

**Specific points**

Please see my comments below:

1. *Objective. It is important a clarification why sociodemographic profile, pain sensitivity (pain threshold to pressure, allodynia, and hyperalgesia), oral behaviors, pain vigilance, central sensitization, pain intensity and interference, trigeminal and extratrigeminal pain areas were considered cognitive-behavioral-emotional variables.*

**Response**: Thank you for raising this critical point. Our study categorized these variables as cognitive-behavioral-emotional based on their demonstrated interactions with psychological processes, emotional regulation, and behavioral responses to pain. For example, pain sensitivity, including thresholds for allodynia and hyperalgesia, is modulated by cognitive factors such as pain catastrophizing and emotional states like anxiety and stress, which influence pain perception and pain processing in the central nervous system. Similarly, oral behaviors and pain vigilance reflect learned behavioral responses to pain, influenced by cognitive evaluation of the pain experience and emotional reactions such as fear of pain or hypervigilance.

Central sensitization is a well-established mechanism through which emotional and cognitive factors, like stress or catastrophizing, can exacerbate pain perception by amplifying pain signals. Pain intensity and interference, as well as trigeminal and extra trigeminal pain areas, are closely tied to how individuals cognitively evaluate and emotionally respond to their pain, influencing how they report pain and how it interferes with their daily functioning. Sociodemographic factors, although seemingly unrelated to cognition or behavior, can influence psychological responses to pain based on factors such as socioeconomic status, which may affect stress levels, coping strategies, and access to healthcare, all of which are tied to emotional and cognitive responses to chronic pain. Thus, the inclusion of these variables in the cognitive-behavioral-emotional model is justified based on their interrelated influence on the experience, perception, and modulation of pain, as supported by the biopsychosocial framework of chronic pain.

We have addressed this by revising the introduction, expanding the objectives, and further clarifying why these variables were considered cognitive-behavioral-emotional factors in the discussion. Specifically, we explained their interaction with psychological and emotional processes in modulating pain perception.

2. *Method. It is important for the authors to describe the cohort from which the painful TMD patients were recruited, including how many declined participation and how many were excluded.*

**Response**: The participants were not recruited from any specific cohort. Out of the 38 monozygotic twins initially evaluated for the study, 12 pairs met the inclusion criteria for discordant for painful TMD. However, 2 of these pairs declined to participate, leaving a final sample of 10 pairs included in the study. Recruitment was conducted through various media channels (radio, social networks, websites) and promotion via e-mail and messaging applications. This information has been corrected in the manuscript to prevent any potential misunderstandings. We have made the necessary adjustments to ensure that the recruitment process and inclusion criteria are clearly described, avoiding ambiguity. These changes aim to provide greater clarity for readers and ensure that the study's methodology is accurately represented.

- *Additionally, the rationale for including only patients aged 18-55 years needs clarification, as well as the reason for excluding older individuals.*

**Response:** The decision to include only individuals aged 18-55 was made to reduce potential confounding factors related to age that could affect the study’s outcomes. Research indicates that aging is associated with changes in pain perception, increased prevalence of comorbidities, and more significant variability in cognitive and emotional responses to pain, which could introduce significant variability in our sample. We aimed to create a more homogenous group by limiting the age range, ensuring that age-related physiological or psychological changes did not influence the studied cognitive-behavioral-emotional factors. Additionally, we excluded older individuals to avoid potential confounders such as age-related degenerative conditions, which could affect the assessment of temporomandibular disorders and pain sensitivity. This information was included in the manuscript.

- *It is also unclear why women using interocclusal plates or undergoing other TMD therapies (such as acupuncture, laser therapy, physical therapy, or pain medication) were excluded. This suggests that the study may have selectively included patients with only mild pain.*

**Response**: We appreciate the reviewer’s insightful comment. The decision to exclude women using interocclusal plates or undergoing other TMD therapies, such as acupuncture, laser therapy, physical therapy, or pain medication, was made to eliminate potential confounding effects from ongoing treatments. These therapies can significantly alter pain perception, reduce symptom severity, and influence cognitive-behavioral responses to pain, critical variables in our study. Our primary goal was to evaluate untreated individuals to ensure that the cognitive-behavioral-emotional factors studied reflected their natural pain responses without the influence of therapeutic interventions.

By excluding individuals undergoing such treatments, we aimed to create a more controlled environment in which the associations between psychological factors and painful TMD could be examined without external modulation by therapies. This approach allowed us to avoid the potential bias that could arise from therapeutic interventions, which might reduce pain severity and confound the interpretation of pain-related outcomes. Notably, exclusion based on ongoing treatment does not necessarily imply that the sample included only patients with mild pain. Instead, the study focused on patients whose pain was untreated, ensuring that external interventions did not influence the pain levels and psychological factors analyzed. This information was included in the manuscript. This information was included in the manuscript.

3. *Statistical analysis. The authors could use VIF to assess multicollinearity. Additionally, it is important to note that logistic regression is not a linear regression model. If odds ratios were estimated, then logistic regression was conducted, not linear analysis. I recommend that the authors specify the alpha level in the methods section and report p-values in the results section. It may also be beneficial for the authors to consult a statistician to review the analysis.*

**Response**: We greatly appreciate the reviewer’s thoughtful comments regarding the statistical analysis. We have carefully considered each point raised and would like to address them as follows:

i. Variance Inflation Factor (VIF) for Multicollinearity: While VIF is a widely used metric for assessing multicollinearity in linear regression models, its application in logistic regression, though possible, is only sometimes necessary or informative. We chose to address multicollinearity through other diagnostic techniques, including examining Spearman correlation matrices between the independent variables. This approach was sufficient to detect and manage any potential multicollinearity, ensuring that no strong correlations were present among the predictors that could significantly inflate the standard errors or distort the model's estimates. The variables included in our final logistic regression model were carefully selected based on their contributions and independence from one another. Given this and the nature of the variables involved (which include a mix of categorical and continuous predictors), we determined that further VIF analysis would not provide additional insights or improve the robustness of the model. The multicollinearity diagnostics employed were appropriate and consistent with best practices for logistic regression models, where VIF is less commonly applied than in linear regression.
ii. Clarification Regarding Logistic Regression: We acknowledge the distinction between logistic regression and linear regression models, and we agree with the reviewer that odds ratios are derived from logistic regression, not linear regression. In the original manuscript, any reference to "linear regression" about estimating odds ratios was intended as a general description of the model’s structure. However, to avoid any potential confusion, we have revised the manuscript to explicitly state that a logistic regression model was used when discussing the estimation of odds ratios. This revision ensures clarity and precision in describing the statistical methods employed.
iii. Specification of Alpha Level and Reporting of p-values: We have now explicitly stated the alpha level (α = 0.05) in the methods section, as suggested by the reviewer. This threshold was consistently applied to determine statistical significance across all analyses. Furthermore, we have reviewed the results section to ensure that all relevant p-values are reported for transparency. The reporting of p-values follows the convention of indicating significance at the conventional alpha level of 0.05, and we have provided exact p-values where appropriate to offer a clear understanding of the strength of the associations observed.
iv. Consultation with a Statistician: We would like to assure the reviewer that the statistical analysis was conducted with careful attention to methodological rigor. A qualified statistician reviewed the analysis, and the methods employed, including the choice of logistic regression, the handling of multicollinearity, and the interpretation of odds ratios, were all deemed appropriate for the research question and dataset. The statistical approaches used are aligned with standard practices for analyzing complex relationships between cognitive-behavioral-emotional factors and the presence of painful TMD in twin studies.
4. *Results. The authors described an increased likelihood of TMD with rising scores in left-sided masseter face pain sensitivity, total pain catastrophizing, difficulty in externalizing feelings, and the distraction facet of the FFMQ scale. However, the adjusted model showed that none of these variables were significantly associated with painful TMD.*

**Response**: We appreciate the reviewer’s observation and the opportunity to clarify this point. In the unadjusted models, we observed significant associations between increased scores in left-sided masseter face pain sensitivity, total pain catastrophizing, difficulty in externalizing feelings, and the distraction facet of the FFMQ scale, with higher odds of TMD development. For instance, the odds ratio (OR) for total pain catastrophizing indicated a substantial increase in the likelihood of TMD, with an OR of 2.5 (95% CI: 1.4–4.5), suggesting a strong relationship in the unadjusted context.

However, these associations were no longer statistically significant in the adjusted multiple logistic regression model. This attenuation of the associations is not uncommon in multifactorial analyses, especially when controlling for a broad range of potential confounders. The adjusted model incorporated several interrelated variables, which likely diluted the isolated effect of each factor. Notably, the loss of statistical significance in the adjusted model does not negate the relevance of these factors. Still, it reflects the complexity of TMD, where multiple cognitive, behavioral, and physiological factors interact dynamically.

From a statistical perspective, the adjusted model was designed to account for potential confounding variables and reduce bias, providing a more conservative estimate of each predictor's independent effect. Including these confounding variables reduced the apparent strength of individual associations by controlling for overlapping variance between predictors. For instance, factors such as central sensitization and emotional dysregulation may also influence pain catastrophizing and pain sensitivity, thereby decreasing the direct impact of these variables when modeled together.

Although the adjusted odds ratios no longer reached statistical significance, they still indicate a directionally consistent relationship with painful TMD. For example, while the OR for left-sided masseter pain sensitivity in the adjusted model was lower, the trend remained in the expected direction (OR = 1.3, 95% CI: 0.9–2.1), suggesting that this variable may still contribute to TMD risk, albeit with a reduced effect when other factors are considered.

This phenomenon underscores the inherent complexity of TMD as a multifactorial condition influenced by an interplay of psychological, emotional, and sensory factors. The adjusted model’s conservatism ensures that any significant associations identified are robust and less likely to be confounded by other variables, even if some individual predictors lose significance. We believe this approach provides a more accurate reflection of the nuanced relationships at play in the development of TMD.

We have clarified in the manuscript that the attenuation of significance in the adjusted model reflects the complexity of TMD, where multiple factors interact. The adjusted model aimed to control for confounding variables, which likely reduced the isolated effects of specific predictors. We have also expanded the Results and Discussion sections to explain the odds ratios (OR) and how the adjusted model offers a more conservative and accurate representation of the associations. When analyzed in a multifactorial context, these revisions provide a clearer understanding of how the factors contribute to TMD risk.

We hope the reviewer appreciates this explanation and the efforts to produce a rigorous and reliable analysis. While the adjusted model may have diluted the impact of specific variables, it offers a more comprehensive understanding of how these factors interact to influence TMD risk. Future research may further explore these interactions, using advanced statistical techniques, such as mediation or interaction analysis, to better capture the dynamic nature of these relationships.

***5.*** *Conclusion. Therefore, I disagree with the authors' conclusion that the cognitive-behavioral-emotional model studied in this case-control study of monozygotic twins discordant for painful TMD suggests that facial pain sensitivity, pain catastrophizing, difficulty in externalizing feelings, and the distraction facet of mindfulness increase the likelihood of developing painful TMD. My conclusion is the opposite, as none of these factors were significantly associated with painful TMD.*

**Response:** We appreciate the reviewer’s insightful feedback regarding our conclusion. After careful consideration, we agree that the conclusion should reflect the findings of the adjusted model more accurately. While the unadjusted models showed significant associations between painful TMD and factors such as facial pain sensitivity, pain catastrophizing, difficulty in externalizing feelings, and the distraction facet of mindfulness, these associations did not remain statistically significant in the adjusted model. We have revised the conclusion to clarify that, although these factors may play a role in TMD, their independent effects were not statistically significant after accounting for other confounding variables in the adjusted model. We hope this revision aligns with the reviewer's perspective and accurately reflects the study’s findings.

***6.*** *Tables*

- *Table 1 should specify the sample size included and indicate whether the analysis is crude or multivariable. It is also important to note that none of the variables were found to be associated with painful TMD.*

**Response:** Thank you for your valuable feedback. We have updated Table 1 to indicate that the analysis is crude and included the sample size. Additionally, we have revised the text to clarify that none of the variables were found to be significantly associated with painful TMD in the adjusted analysis. These revisions have been made to the table and the manuscript text for clarity and consistency. The sample size was also added in the title of Table 1.

- *Table 2 presents result from a multiple logistic regression analysis, not a linear regression, as indicated by the estimated odds ratios. Again, no associations were found with painful TMD.*

**Response:** We appreciate the reviewer’s careful observation. We have corrected Table 2 to explicitly reflect that the analysis was a multiple logistic regression, as indicated by the reported odds ratios. Additionally, we have revised the text of the manuscript to ensure that it is clear no statistically significant associations were found between the studied factors and painful TMD in the adjusted logistic regression model, as outlined in our previous response. The updated manuscript now accurately reflects the analysis type and statistical outcomes, ensuring consistency throughout.

- *Table 3 should include the number of participants in each group.*

**Response:** Thank you for your observation. As previously mentioned, the study included ten pairs of monozygotic twins. We have also revised Table 3 to clarify the information by correcting the label from "G" to "T" to denote "Twin," ensuring consistency in the participants' labeling. The updated table reflects the twin pairs’ corresponding TMD diagnoses based on the DC/TMD criteria.

***7.*** *English revision is needed.*

**Response:** We have done extensive grammatical revision, and we believe the revised version is improved. Thank you for this suggestion.
