# Supplementary material for: Phenotypes of painful TMD in discordant monozygotic twins according to a cognitive-behavioral-emotional model: a case-control study
Source: PLoS One. 2025 Apr 17;20(4):e0320515. doi: 10.1371/journal.pone.0320515 (PMC12005551; doi:10.1371/journal.pone.0320515)
Supplement: Attachment — Submitted filename: Response_to__Reviewers_auresp_2.docx [file pone.0320515.s003.docx]

**Academic Editor – PLOS ONE	Manuscript PONE-D-24-27113R1**

After reviewing the article, important revisions were identified to enhance the clarity and consistency of the manuscript before it is ready for publication. Key points include the need for language editing, adjustments to the abstract to better clarify the objectives, methodology, and clinical relevance, as well as greater precision in the introduction and conclusion to align them with the results. In the methodology, more detailed information on the recruitment process and clear justifications for exclusions and analyses performed were requested. Additionally, inconsistencies in the statistical approach were highlighted, with a recommendation for review by a statistician to ensure the appropriateness of the model and interpretations.

**Reviewer #2	Manuscript PONE-D-24-27113R1**

**This study addresses an interesting topic; however, the manuscript requires significant revisions.**

RESPONSE: Thank you for your feedback and for recognizing the relevance of our study topic. We have carefully reviewed the manuscript and made significant revisions to address the concerns raised. These include improving the clarity of the abstract, refining the methodology section, ensuring consistency between the results and discussion, and revising the language for better readability. We are confident that these changes enhance the quality and scientific rigor of the manuscript.

**1. English Language: A thorough review and revision of the English language are necessary to improve clarity and readability.**

RESPONSE: Thank you for your observation. A thorough review and revision of the manuscript have been conducted by a native English speaker with expertise in academic writing to ensure clarity and improve readability throughout the text.

**2. Abstract:
a. The abstract lacks clarity. The aim and methodology are not clearly articulated. For instance, it is unclear how the 20 monozygotic twins with discordant conditions were selected.**

RESPONSE: Thank you for your observation. We have revised the abstract to enhance clarity regarding the aim and methodology. Specifically, we have included a concise description of the recruitment process for the 20 monozygotic twins discordant for painful TMD, detailing the comprehensive strategy employed to identify and include eligible participants. We hope these adjustments address the reviewer’s concerns and improve the overall clarity of the abstract.

**b. Conclusion and Clinical Relevance are wrong.**

RESPONSE: These topics were rewritten.

“**Conclusions:** Our study highlights the potential clinical relevance of cognitive-behavioral-emotional variables, such as increased pain sensitivity in the masseter muscle, higher levels of pain catastrophizing, difficulty in externalizing feelings, and higher scores on the distraction facet of mindfulness, in understanding painful TMD. While these variables did not show statistical significance in the adjusted model, their inclusion underscores the importance of exploring these factors in clinical practice. Further research is needed to validate these findings and clarify their role in the development and management of painful TMD.. **Clinical Relevance:** This study underscores the importance of cognitive-behavioral-emotional factors in the context of painful TMD, suggesting that variables like pain sensitivity and emotional regulation may be valuable for clinical assessment and management strategies. Despite the lack of statistically significant associations, these findings provide a foundation for future research to better understand and address the multidimensional nature of TMD in clinical practice.”

**c. Presentation: The frequent use of nested parentheses is not appropriate (e.g., (OR=3.29; 95% CI=(0.17–62.8), p=0.428)). Please revise to ensure a cleaner and more professional presentation.**

RESPONSE: Thank you for your observation. We have revised the abstract to remove nested parentheses, ensuring a cleaner and more professional presentation. For instance, "95% CI= (0.17–62.8)" was reformatted to "95% CI: 0.17–62.8." Similar adjustments have been made throughout the text to maintain consistency.

**3. Introduction: Please note that self-reported parafunctions do not fall under the category of "orofacial symptoms, the frequency of somatic symptoms, poor sleep quality, and genetic and epigenetic factors." This distinction should be clarified in the introduction.**

RESPONSE: The distinction regarding self-reported parafunctions has been clarified in the introduction to ensure alignment with the context of the predictors for painful TMD.

**4. Method:
a. Suggestion: Begin by clearly describing the recruitment process for the study participants. Following this, provide details about the sample: 38 monozygotic twins were initially evaluated, and among them, 12 pairs met the inclusion criteria for discordance in painful TMD.**

RESPONSE: We fully understand and appreciate your concern regarding the detailed description of the recruitment process, as this was one of the most challenging aspects of conducting this study. During the conceptualization phase, we explored multiple approaches to identify suitable participants. Given the specificity of the condition under investigation (TMD is not widely recognized in the medical field), recruiting monozygotic twins with discordance in painful TMD presented unique challenges. To overcome these difficulties, we implemented a comprehensive recruitment strategy. After obtaining ethical approval, we disseminated information about the study and its inclusion criteria through various channels, including social media platforms, the websites of FORP/USP and FFCLRP/USP, USP Radio and local radio stations in Ribeirão Preto and around, messaging apps targeting student and community groups, and physical posters placed in strategic locations throughout Ribeirão Preto. These efforts resulted in 38 twin pairs contacting our research team, all of whom were scheduled for clinical evaluations. Among these, 12 pairs met the inclusion and exclusion criteria, and 10 pairs agreed to participate in the study. The extended data collection period reflects the significant challenges associated with recruiting such a specific and unique sample. We have incorporated these details into the manuscript to enhance transparency and address your concerns regarding the recruitment process.

**b. Age Inclusion Criterion: The authors justify including only individuals aged 18–55 to reduce potential age-related confounding factors. While this is a valid rationale, it should also be acknowledged as a limitation. Age could have been accounted for as a potential confounder in the analysis.**

RESPONSE: We agree with this limitation. We have acknowledged this in the discussion, noting that age could have been treated as a potential confounder in the analysis to provide a broader understanding of its influence. This adjustment enhances the transparency and depth of our study's limitations.

**c. Statistical Model Explanation: Upon reviewing the explanation of the statistical model, it appears the authors may not have fully understood the initial comment, as their response does not align with the original concern.**

RESPONSE: All statistical analyses in this study were conducted by a highly qualified professional statistician with a Master’s and Ph.D. in statistics, specializing in the design and analysis of clinical research. Furthermore, all adjustments to the manuscript and responses to this review have been carefully developed in collaboration with this expert. We greatly value the reviewer’s thoughtful concern regarding the statistical aspects of the study and are diligently working to address and incorporate the insightful suggestions provided. We would also like to respectfully acknowledge the inherent limitations of our study, particularly related to the unique nature of the sample—monozygotic twins discordant for painful TMD—which required the statistical models to be tailored to this specificity. We deeply appreciate the reviewer’s input and are committed to improving the manuscript to ensure it meets the highest standards of scientific rigor.

**If the adjusted model includes variables that are not statistically significant, the authors must explain the implications of these findings, particularly by referencing the 95% confidence intervals as a guide. Again, I strongly suggest consulting a statistician to address these issues thoroughly.**

RESPONSE: The reviewer is correct to highlight the wide confidence intervals and the high degree of uncertainty they reflect regarding the effect estimates for the variables *Pain sensitivity - Face, left, masseter* and *FFMQ - Distraction Facet Score*, as presented in Table 2. We fully acknowledge the uncertainty associated with these effects. However, we believe that these variables may represent important characteristics (signs/symptoms) relevant to clinical practice. It is within this context that we emphasized their discussion in the manuscript, aiming to draw attention to their potential relevance despite the statistical limitations. We appreciate the reviewer’s thoughtful observation and have included additional clarification in the discussion to better address these points.

**Multicollinearity: I disagree with the approach used to assess multicollinearity. Variance Inflation Factor (VIF) should be the primary method, as it evaluates the combined effect of all predictors and provides a direct measure of their impact on the regression model. Correlation analysis can be used as a complementary tool to identify pairwise relationships that may warrant further investigation or justify the use of VIF. Clarify whether collinearity was assessed using logistic regression models and provide an accurate description of the statistical approach.**

RESPONSE: We acknowledge VIF as a useful measure for detecting multicollinearity. However, we chose to investigate multicollinearity by examining the correlation matrix among the independent variables because we wanted to avoid the effect of overestimating the multiple determination coefficient (R²), given that many independent variables presented p < 0.20 in the bivariate analysis. Furthermore, the literature provides various analogs to R² in the context of logistic regression, which led us to question whether R² would be appropriate in this context. If R² is not appropriate, then VIF = 1/(1-R²) would also not be suitable. Considering this, we opted to examine the correlation matrix among the independent variables, which is also a valid method for investigating multicollinearity.

**To address potential confounding (line 16, page 7), the authors could adjust the analysis rather than relying on exclusions, which should only be employed if there are very few cases within the excluded group. Additionally, the authors should list the number of subjects excluded in each group.**

RESPONSE: From an analytical perspective, the exclusions (related to interocclusal appliances, alternative therapies, neurological disorders, psychiatric disorders, and pregnancy) were necessary because, when combined, they would generate numerous binary variables, some with low prevalence. Given the sample size, any statistical technique applied would produce effect estimates for these variables with low precision. From a clinical perspective, the exclusions were essential as we aimed to focus on cognitive-behavioral-emotional factors. The number of subjects excluded was included in the Methods section.

**Statistical Analysis: This section requires verification by a statistician, as it is confusing and contains inaccuracies. For instance, the statement, "The association of each factor with TMD was investigated by fitting pairwise conditional simple logistic linear regression models," is problematic. Is the analysis "simple"? Are "logistic" and "linear" being used interchangeably?**

RESPONSE: Yes. In the bivariate phase, each independent variable is individually associated with the outcome. In statistics, when the outcome variable is associated with a single independent variable through a regression model, this model is referred to as simple regression. Furthermore, since our outcome is binary (presence of TMD / absence of TMD), the regression used was logistic regression. Additionally, logistic regression is one of several regressions within the class of generalized linear models and can therefore be considered linear because the functional relationship of each independent variable with the outcome is assumed to be linear.

**Why was linear regression used (line 28, page 9)? The authors need to specify the dependent variable to justify the choice of this statistical method.**

RESPONSE: We adopted a linear regression model because we assumed that the functional relationship of each independent variable with our outcome is linear.

**The explanation of the four excluded variables is unclear. This section reads more like results than methodology. For example, the finding of a borderline association between trapezius pain threshold and TMD (OR = 0.29, 95% CI = 0.07–1.20, p = 0.088) raises questions—why was this variable excluded despite its potential relevance? The authors should provide a clear rationale for this decision.**

RESPONSE: Despite their apparent potential, some variables, including the one mentioned by the reviewer, were excluded due to moderate to high correlation with other equally important variables.

**The sentence, “Although these variables showed associations with TMD in the bivariate analysis, their high correlation with other variables in the model necessitated their exclusion,” is unclear and problematic.**

RESPONSE: Our justification lies in the pursuit of a parsimonious final model that includes, in its deterministic component, variables with the lowest possible degree of correlation among them.

**Are the authors excluding variables that are associated with TMD?**

RESPONSE: Due to the small sample size, if all independent variables with p < 0.20 (Table 1) were included in the multiple regression analysis shown in Table 2, the logistic regression model would have extremely low power. For this reason, we used the correlation between the independent variables with p < 0.20 to include in the model presented in Table 2 only the variables that were the most independent from one another.

**This contradicts the study's objective.**

RESPONSE: We understand that this may seem contradictory, but a good model must also provide precise estimates and be parsimonious. Given the conditions (many independent variables with p < 0.20 eligible for the final model and a small sample size), prioritizing parsimony and selecting the most independent variables was necessary for the screening process.

**I strongly recommend conducting a principal component analysis (PCA) to address multicollinearity and using the resulting components in the logistic regression instead of excluding potentially relevant variables.**

RESPONSE: The use of PCA would reduce the independent variables to a small set of principal components with a certain degree of orthogonality between them, but it would introduce the disadvantage of interpreting the effects of each principal component on our outcome.

**5. Results.
a. The sentence, "The participants were not recruited from any Brazilian-specific cohort," should be deleted. Before discussing the evaluation of the 38 monozygotic twins, the authors need to clearly explain the recruitment process of the participants.**

RESPONSE: The sentence was excluded, and the recruitment process was better explained.

**b. The statement, “The variables that showed a positive association (according to the OR), however, without statistical significance, were: pain duration…” is incorrect. What is the risk of a Type I error here? Focus on interpreting the 95% confidence intervals (CIs) and p-values.**

RESPONSE: Considering the reviewer’s recommendations, the estimation of type I errors for each association is indeed high. For this reason, we recognize that using phrases such as “positive association, but not statistically significant” may seem inconsistent. To address the reviewer’s suggestions, we have revised our interpretation of the associations in the final model, focusing on confidence intervals. Based on these intervals, the most precise associations were observed with the variables *Pain Catastrophizing - Total score* and *Difficulty in Expressing Feelings Score*. However, the variables *Pain sensitivity - Face, left, masseter* and *FFMQ - Distraction Facet Score* also hold clinical relevance. Nevertheless, it is important to note that all these variables showed non-significant associations. Adjustments in the text were made to correct this.

**6. Discussion: The sentence, “Our findings revealed that increased pain sensitivity in the masseter muscle, higher levels of pain catastrophizing, difficulty in externalizing feelings, and higher scores on the distraction facet of mindfulness were significant predictors of painful TMD. These results provide evidence that, even in genetically identical individuals, cognitive and emotional factors play a crucial role in the manifestation of chronic pain associated with TMD” (lines 6–10, page 16), is incorrect and does not align with the study's findings. This discussion must be revised to accurately reflect the results. The claims about significant predictors and their implications should only be made if they are strongly supported by the analysis.**

RESPONSE: The sentence was revised.

“Our findings indicate that increased pain sensitivity in the masseter muscle, higher levels of pain catastrophizing, difficulty in externalizing feelings, and higher scores on the distraction facet of mindfulness were included in the final model due to their clinical relevance and their associations in the bivariate analysis (p < 0.20). Despite their lack of statistical significance in the adjusted model, these variables were selected to ensure a comprehensive exploration of cognitive-behavioral-emotional factors in painful TMD, particularly given their potential importance in clinical practice. The inclusion of these variables also reflects our effort to balance parsimony with the need to retain independent predictors while considering the limitations imposed by the sample size and the specificity of the study population.”

**7. Conclusion: The conclusion is not consistent with the findings. The results indicate that the variables analyzed are not significantly associated with TMD and do not suggest any interaction. Instead, the findings highlight that the crude analysis was likely confounded. The conclusion should be rewritten to reflect these points accurately.**

RESPONSE: Thank you for your insightful feedback. We have carefully reviewed your comments and made significant efforts to revise the manuscript to incorporate your suggestions. We have adjusted the discussion and conclusion sections to better reflect the study’s findings and align with the data presented. The inclusion of specific variables in the final model has been clarified, emphasizing their clinical relevance and the rationale based on the bivariate analysis. We hope that these revisions address your concerns and improve the clarity and scientific rigor of the manuscript. We are grateful for your valuable input and remain open to further suggestions.

“Our study investigated cognitive-behavioral-emotional factors potentially associated with painful TMD in a unique sample of monozygotic twins discordant for the condition. While the adjusted model did not identify statistically significant associations, variables such as increased pain sensitivity in the masseter muscle, higher levels of pain catastrophizing, difficulty in externalizing feelings, and higher scores on the distraction facet of mindfulness were included due to their clinical relevance and their significant associations in the bivariate analysis. These findings contribute to understanding the potential role of these variables in painful TMD, highlighting their relevance for clinical practice and the need for further exploration. Our approach emphasizes the importance of balancing parsimony and clinical applicability, while recognizing the limitations of sample size and study specificity. Future studies with larger populations are essential to confirm these results and to further investigate the interplay between cognitive-behavioral-emotional factors and painful TMD.”

**Reviewer #3	Manuscript PONE-D-24-27113R1**

**The introduction aligns well with the research objectives, emphasizing the biopsychosocial model and the complex interactions between genetic, emotional, and behavioral factors. The particularly relevant references to twin studies genetic mechanisms and the role of genetics and epi strengthen the justification for the study design.**

RESPONSE: Thank you for your positive feedback.

**The discussion explores how cognitive, behavioral, and emotional dimensions interact with pain perception in TMD, reinforcing the biopsychosocial model. The findings, including the role of pain catastrophizing, emotional regulation, and mindfulness, provide actionable insights for developing targeted interventions.**

RESPONSE: Thank you for your comment. We are pleased that the discussion underscores the relevance of cognitive, behavioral, and emotional factors in TMD and supports actionable strategies like mindfulness and emotional regulation for targeted interventions.

**The study effectively bridges psychosocial theories with clinical practice, providing a multidimensional understanding of TMD. Its innovative design, focusing on monozygotic twins, offers unique insights into genetic and environmental contributions. Future work should aim to validate these findings in diverse and larger populations, using more sophisticated statistical models to unravel the intricate web of influencing factors.**

RESPONSE: Thank you for your insightful feedback. We are pleased that the study's integration of psychosocial theories with clinical practice and its focus on monozygotic twins were recognized as innovative. We agree that future research with larger, more diverse samples and advanced statistical models will be essential to further validate and expand upon these findings.
